# Deficiency of Adiponectin Protects against Ovariectomy-Induced Osteoporosis in Mice

**DOI:** 10.1371/journal.pone.0068497

**Published:** 2013-07-02

**Authors:** Fang Wang, Pei-xia Wang, Xiao-lin Wu, Su-ying Dang, Yan Chen, Ying-yin Ni, Li-hong Gao, Shun-yuan Lu, Ying Kuang, Lei Huang, Jian Fei, Zhu-gang Wang, Xiao-fen Pang

**Affiliations:** 1 Department of Medical Genetics, E-Institutes of Shanghai Universities, Shanghai Jiao Tong University School of Medicine (SJTUSM), Shanghai, China; 2 Department of Geriatrics, Ruijin Hospital affiliated to SJTUSM, Shanghai, China; 3 Shanghai Research Center for Model Organisms, Shanghai, China; Oklahoma State University, United States of America

## Abstract

Adipokine adiponectin (APN) has been recently reported to play a role in regulating bone mineral density (BMD). To explore the mechanism by which APN affects BMD, we investigated BMD and biomechanical strength properties of the femur and vertebra in sham-operated (Sham) and ovariectomized (OVX) *APN* knockout (KO) mice as compared to their operated wild-type (WT) littermates. The results show that APN deficiency has no effect on BMD but induces increased ALP activity and osteoclast cell number. While OVX indeed leads to significant bone loss in both femora and vertebras of WT mice with comparable osteogenic activity and a significant increase in osteoclast cell number when compared to that of sham control. However, no differences in BMD, ALP activity and osteoclast cell number were found between Sham and OVX mice deficient for APN. Further studies using bone marrow derived mesenchymal stem cells (MSCs) demonstrate an enhanced osteogenic differentiation and extracellular matrix calcification in *APN* KO mice. The possible mechanism for *APN* deletion induced acceleration of osteogenesis could involve increased proliferation of MSCs and higher expression of *Runx2* and *Osterix* genes. These findings indicate that APN deficiency can protect against OVX-induced osteoporosis in mice, suggesting a potential role of APN in regulating the balance of bone formation and bone resorption, especially in the development of post-menopausal osteoporosis.

## Introduction

Fat mass is closely associated with both bone turnover and density. Adiponectin, the most abundant protein derived from the adipocytes [1], has been reported to contribute to this relationship. Previous studies have suggested that APN and its receptors are expressed in bone-forming cells [2]. However, the manner in which APN affects bone formation remains unclear both *in vivo* and *in vitro*. Some studies reveal that human osteoblasts treated with APN display an increased proliferation, differentiation and increased mineralization, suggesting a positive effect of APN on bone formation [3], [4]. While the bone marrow cells isolated from APN-deficient mice have reduced osteogenesis as compared to WT mice [5]. In addition, C57BL/6J mice treated with an adiponectin-adenovirus for 2 weeks show an increase in trabecular bone, a decreased number of osteoclasts and reduced circulating N-telopeptides of type collagen (NTX) as compared to LacZ-adenovirus-treated mice [6]. These results suggest that APN positively regulates bone mass.

However, other studies suggest adiponectin seems to exert a negative net effect on bone mass and to be an independent predictor of lower bone mass [7]–[12]. Clinical studies show that an increasing level of APN is associated with a significantly decreased whole body bone mineral content (BMC) and BMD, indicating a negative association between APN and BMD [7], [8]. Studies with mouse models have further confirmed the role of APN in negative regulation of BMD. The data from *APN* transgenic mice indicate that the elevated circulating APN could inhibit the acquisition of bone mass in growing mice and result in decreased biomechanical measures of functional strength that are surrogate measures of susceptibility to fractures [9]. In agreement with these results, trabecular bone volume and trabecular number are increased at 14-week of age by 30% and 38% in *APN* KO male C57BL/6J mice, respectively [10]. Meanwhile, another study using 8-week old transgenic male mice in which *APN* is specifically expressed in the liver reveals the differences neither in BMD of femur, tibia or vertebrae, nor in bone formation or resorption parameters as compared to their WT littermates [13].

In order to investigate the function of APN in bone metabolism, we generated *APN* KO mice [14], [15]. Although we did not find any significant differences in bone formation between the two genotypes under physiological conditions, we believed that APN would play an important role in some pathological processes related to bone metabolism. In this study, we measured BMD and biomechanical strength properties of femur and vertebra in ovariectomized and sham-operated *APN* KO mice as compared to their operated WT littermates. We also evaluated the proliferation, differentiation, and mineralization ability of the MSCs from *APN* KO and WT mice. Our data indicate that APN deficiency suppresses bone loss under the condition of ovary removal, probably as a result of increased proliferation of MSCs and higher expression of *Runx2* and *Osterix* genes.

## Materials and Methods

### Animal Studies

All procedures were approved by the Animal Care and Use Committee of Shanghai Jiao Tong University School of Medicine. The *APN* KO mouse model in which the *APN* gene is null was generated previously in our lab [14], [15]. Briefly, a targeting vector was constructed by replacing the mouse *APN* genomic fragment covering exon2 and exon3, with the pGK-neo gene cassette. Genomic DNA from mouse tails was used for genotyping by PCR. The mRNA expression level of *APN* was identified using RT-PCR and its protein level was detected using an APN ELISA kit (RayBiotech Inc., Norcross, GA). The primer sequences are listed in Table 1. Congenic C57BL/129S1 *APN* KO and WT mice were housed under l2-h light/12-h dark cycles in a controlled environment with 40–50% relative humidity at 22°C. All experimental procedures were conducted in accordance with National Institutes of Health Guide for the Care and Use of Laboratory Animals. Mice were individually caged, given free access to regular water and chow (Shanghai SLRC laboratory animal LLC, Shanghai, China, national standard number of laboratory animals’ nutrients for formula feeds is GB 14924.3-2010) before and after ovariectomy surgery. Age-matched (6–8 weeks of age) KO female mice and their WT littermates were weighed and then underwent OVX or sham-operation. The number of mice in WT.Sham, WT.OVX, KO.Sham and KO.OVX was 5, 6, 6, and 7, respectively. All surgeries were performed by one person under sodium pentobarbital anesthesia, and all efforts were made to minimize animal suffering and to reduce the number of animals used. 10 weeks post-surgery, mice were weighed again and then euthanized by cervical dislocation. The left femora and the L2–5 vertebrae with the skin and muscle removed were kept in physiological saline and stored at −20°C for detection of BMD and for biomechanics procedures. For protein and RNA analysis, the right femora, L1 vertebrae and tibiae were frozen in liquid nitrogen, and stored at −80°C until use.

**Table 1:** List of the primers used in the study.

Productc	Sequence	Size (bp)
*WT allele 1*	Up: GAGAGTCCTGAGTATTATCCACACG	4540
	Low: TATTTTTTACTGCCGCTATCTGG	
*MT allele 1*	Up: GCTACCGGTGGATGTGGAAT	3680
	Low: CTGCTCTTGCCCCAAACTGA	
*WT allele 2*	Up: GTAGATGCAGATCTTTGGAGTGGA	691
	Low: AATCATGGTACTGAGTGCTTTAGAA	
*MT allele 2*	Up: TGGGGATTGGAGAGATGGCTTA	904
	Low: TTTGAGGGGACGACGACAGTATC	
*β-actin*	Up: ACGGCCAGGTCATCACTATTG	351
	Low: CCTGCTTGCTGATCCACATCT	
*APN*	Up: ACCAGGCCGTGATGGCAGAGAT	839
	Low: TGTGAAGCCCCCATACCAAATGTG	
*ALP*	Up: TCGGGACTGGTACTCGGATAA	779
	Low: CTGGTAGTTGTGAGCGTAAT	
*OC*	Up: CTCTGTCTCTCTGACCTCACAG	360
	Low: GGAGCTGCTGTGACATCCATAC	
*Col1α*	Up: GAGGCATTAAGGGTCATCGTGG	761
	Low: CATTAGGCGCAGGAAGGTCAGC	
*Osterix*	Up: GGCAAGAGGTTCACTCGTTC	497
	Low: GTCTGACTGGCCTCCTCTTC	
*RUNX2*	Up: GAGGTACCAGAT GGGACTGTG	103
	Low: TCGTTGAACCTTGCTACTTGG	

### Measurement of Bone Mineral Density by DEXA

Bone mineral density (BMD, mg/cm^2^) was measured for the left femora and L2–5 vertebrae by dual-energy X-ray absorptiometry (DEXA) using a PIXImus Instrument (GE Lunar, Madison, WI).

### Bone Biomechanical Testing

Prior to biomechanical testing, BMD of the femora and L2–5 vertebrae was measured, as described above. Then the femora were used for the three-point bend testing that was performed by a computer-controlled mechanical testing machine (Instron-5543, USA) equipped with a 500N M-SI sensor (Celtron) under the following conditions: sample space, 9 mm and plunger speed, 1.8 mm/min at room temperature. The load-deformation curve was plotted, and based on this curve, the bone biomechanical indices, including the energy, maximum load and maximum stress were calculated. The L3 vertebra was loaded to failure by compression testing using the same equipment. The parameters were obtained via a computer-assisted analyzer installed in the load cell.

### Bone Histomorphometry and Histological Analysis

The left femora and L1 vertebrae with the skin and muscle removed were fixed in 10% formaldehyde for a week. Then they were rinsed with ddH_2_O and decalcified in 10% EDTA-2Na in 0.1 M Tris buffer (pH 7.3) for 4 weeks at room temperature. After washed with phosphate buffer, the decalcified specimens were then embedded in paraffin and sectioned with a microtome. H&E staining was performed to study the histological changes. Tartrate-resistant acid phosphatase, a marker enzyme for osteoclasts, was detected using enzyme histochemistry with naphthol AS-MX phosphate (Sigma–Aldrich Corp.).

### ALP Activity Assay

Frozen L1 vertebrae were homogenized in 0.5% NETN buffer containing protease inhibitor cocktail (Sigma, St. Louis, MO, USA) with a homogenizer at 4°C. After centrifuging at 12,000 g for 10 minutes at 4°C, the supernatant protein was quantified using BCA protein assay reagent (Pierce, Rockford, IL). ALP activity was quantified using an ALP detection kit (Nanjing Jiancheng Biotechnology Institute, Nanjing, China) and a spectrophotometer (Bio-Rad, Hercules, CA, USA) at a wavelength of 520 nm. Each value was normalized against the protein amount in the reaction, and expressed as “U/gprot”, according to the manufacture’s instruction.

### Semi-quantitative RT-PCR Analysis

Differential gene expression was detected by semi-quantitative RT-PCR. Total RNA was isolated from frozen lumbar using the TRIzol reagent according to the manufacturer’s protocol (Invitrogen, Carlsbad, CA, USA). Semi-quantitative RT-PCR was performed with cDNA reverse-transcribed from 1 µg of total RNA using AMV reverse transcriptase (Takara, Otsu, Japan). The cycle threshold values were normalized to the expression of the housekeeping gene *β-actin*. Band density was scanned and calculated.

### Bone Marrow Cell Cultures

The number of adherent mesenchymal stem cell (MSC) colonies was determined by the following established procedures [16]. Briefly, bone marrow cells were collected from the femora and tibiae of 8-week-old *APN* KO female mice and WT littermates. For colony-forming assays, cells were plated at a density of 5×10^6^ cells/well in a 12-multi-well plate and cultured for 8 days in α-MEM (Gibco, CA, USA) containing 10% FBS, 100 units/ml penicillin and 100 µg/ml streptomycin. Media was changed every third day. On day 8, cells were fixed with 4% paraformaldehyde, stained with 0.5% crystal violet in 4% paraformaldehyde. The number of colonies was then counted. Colonies of less than 2 mm in diameter and faintly stained colonies were ignored [17]. MSC proliferation assay was performed by cytometry. Cells with different genotype were plated at same density in a 12-multi-well plate. On days 6, 7 and 8, cells were collected by trypsin digestion and cell number was counted using a Beckman Coulter Vi-cell XR Cell Viability Analyzer.

### *In vitro* ALP Activity and Ca^2+^ Content

For ALP and Ca^2+^ analysis, cells were plated at a density of 2×10^7^ cells/well in a 6-multi-well plate in α-MEM containing 10% FBS. After 8 days of culture, cells were washed twice with PBS and lysed in 0.5% NETN buffer containing a protease inhibitor cocktail (Sigma). ALP activity and Ca^2+^ content in the lysate were measured using different kits (Jiancheng Technology) and the protein content was determined using BCA protein assay reagent (Pierce Biotechnology). ALP activity and Ca**^2+^** content were measured using certain amount of protein and normalized against the protein amount in the reaction, and expressed as “U/gprot” and “mmol/gprot”, respectively.

For the analysis of osteogenesis, bone marrow cells were plated at a density of 5×10^6^ cells/well in a 12-multi-well plate in α-MEM containing 10% FBS and cultured for 8 days. Cells were further induced for 3 days with added ascorbic acid, dexamethasone and β-glycerophosphate. On day 12, culture plates were rinsed with PBS, fixed with 4% paraformaldehyde, and stained with 2% Alizarin red S (pH 4.0) (Sigma) [5]. For mRNA assays, cells were plated at a density of 2×10^7^ cells/well in a 6-multi-well plate and cultured for 8 days in α-MEM containing 10% FBS. Total RNA was extracted from the cultures, using the TRIzol reagent RNA extraction protocol according to manufacturer’s instructions (Invitrogen). Semi-quantitative RT-PCR was performed by using cDNA reverse-transcribed from 1 µg of total RNA. PCR cycles varied from 26 to 34 cycles. Band density was scanned and calculated.

### Statistical Analysis

The results are expressed as mean±standard deviation (SD) from at least 3 independent experiments. The statistical analysis was performed using Student t test or analysis of variance (ANOVA) with post hoc Scheffe testing when appropriate (SPSS v19). P<0.05 was considered statistically significant.

## Results

### Deficiency of APN Inhibits OVX-induced Osteoporosis in Mice

**Figure 1:**
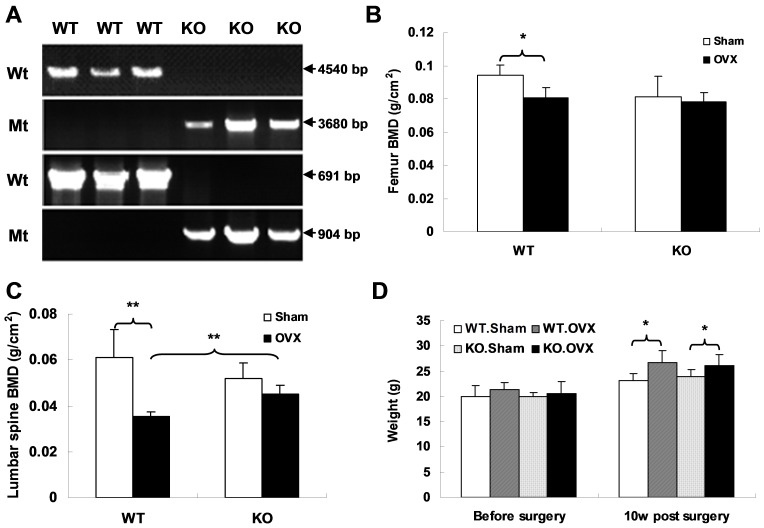
OVX results in significant bone loss in WT but not *APN* KO mice. (A) Two-pair primers were used for PCR genotyping. The products derived from both WT and targeted alleles were amplified with expected size, respectively. (B, C) BMD of femora and L2–5 vertebrae was evaluated by the DEXA method (WT.Sham, n = 5; WT.OVX, n = 6; KO.Sham, n = 6; KO.OVX, n = 7). (D) Change in body weight among the four groups was assessed either before or 10 weeks post surgery. Data is expressed as mean ± SD. *indicates *p*<0.05, **indicates *p*<0.01.

The *APN* KO mouse model was generated by the homologous recombination method. Exons 2 and 3 of the *APN* gene were replaced with the pGK-neo gene. Genomic DNA from mouse tails was used for genotyping by PCR reactions with two different pairs of primers (Figure 1A). To investigate the role of APN in bone metabolism, we analyzed BMD in *APN* KO mice, as well as in their age- and sex-matched WT littermates. We found that there is no significant difference in BMD between *APN* KO and WT mice (data not shown). We therefore performed OVX or sham surgery when the mice were 8 weeks old. 10 weeks after surgery, several indices were measured to evaluate the bone metabolism upon OVX between two different genotypes of mice. We found that the BMD of both femora and vertebrae decreased significantly in WT mice after OVX surgery as compared to those with sham surgery (*p*<0.05, Figure 1B, 1C). However, no significant differences were found in either femora or vertebrae of the *APN* KO mice between OVX and sham surgery groups (p>0.05, Figure 1B, 1C). To test whether OVX has any effect on body weight, *APN* KO and WT mice were weighed before and 10 weeks post surgery. The results show that no differences were found in body weight between *APN* KO and WT mice before OVX surgery, but body weight was augmented after OVX surgery both in WT and KO mice (WT.OVX vs WT.Sham = 26.6±2.45 g vs 23.12±1.49 g, *p* = 0.035; KO.OVX vs KO.Sham = 26.68±1.80 g vs 23.83±1.56 g, *p* = 0.018, Figure 1D).

Biomechanical properties of *APN* KO mice and their WT littermates were further measured by a three-point bending test and a compression test. We found there were no differences of energy, maximum load or maximum stress of vertebrae and femora between two genotypes under physiological conditions with sham operation. Following OVX, WT mice showed a significant reduction in these biomechanical parameters (Table 2), indicating that bone strength in WT mice was reduced because of the surgery. However, OVX treatment did not result in statistically significant differences in these parameters in *APN* KO mice, as compared with sham operated mice (Table 2). This result indicates the protection of bone strength by APN deletion upon OVX surgery. Taken together, these results demonstrate that WT mice, but not KO mice have an osteoporosis phenotype after OVX. In other words, APN deficiency significantly inhibits OVX induced osteoporosis development.

**Table 2:** Biomechanical parameter changes in *APN* KO and WT mice upon OVX surgery.

	Energy (mJ)		Maximum Load(N)		Maximum Stress (Mpa)	
	lumbar	femur	lumbar	femur	lumbar	femur
WT.Sham	24.39±2.49	4.45±0.13	50.69±10.50	23.33±0.90	0.59±0.01	105.03±5.41
WT.OVX	14.24±2.82**	3.06±0.18**	28.69±4.67**	17.53±1.99**	0.38±0.04**	92.87±9.60*
KO.Sham	20.92±8.04	3.31±0.53	47.74±2.53	21.04±0.79	0.64±0.06	160.25±10.07
KO.OVX	20.53±6.64	3.48±0.67	42.07±4.11	22.62±3.04	0.54±0.05	146.73±1.86

Note: Mean values ± SD are shown. WT.Sham, n = 5; WT.OVX, n = 6; KO.Sham, n = 6; KO.OVX, n = 7.

* indicates significant differences between WT.OVX and WT.Sham mice at *p*<0.05,

** indicates significant differences between WT.OVX and WT.Sham mice at *p*<0.01.

**Figure 2:**
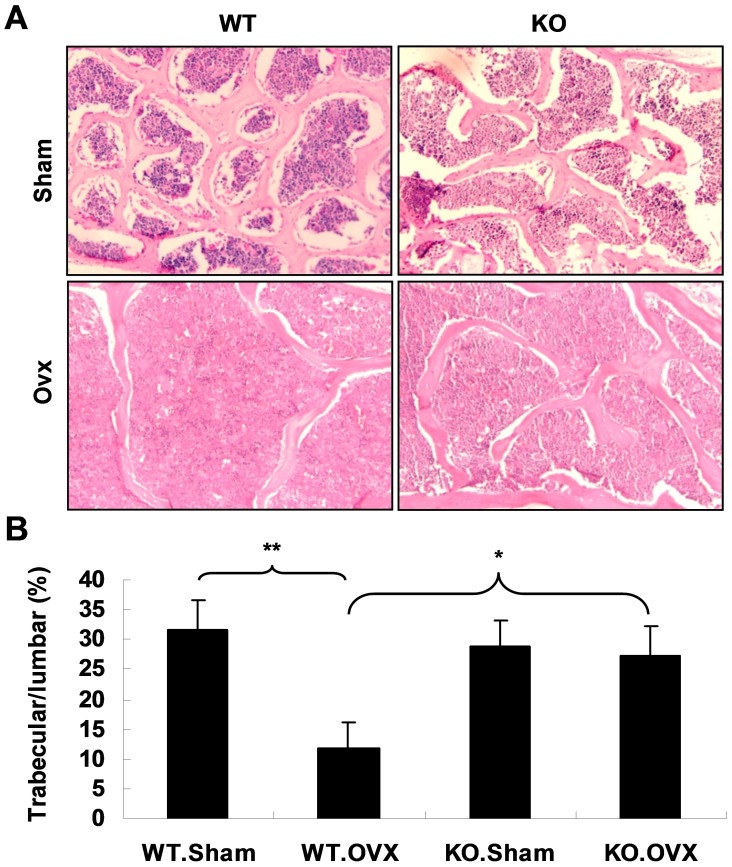
OVX surgery leads to dramatically decreased bone trabeculae in WT but not in *APN* KO mice. (A) H&E staining on the sections of L1 vertebra shows significant reduced bone trabeculae in WT mice upon OVX, but not in *APN* KO mice. Original magnification 100×. (B) The ratios of bone trabeculae to vertebra area were calculated. The values are presented as mean ± SD (n = 3). Trabecular/lumbar means the ratio of bone trabeculae proportion/area to vertebra proportion/area. *indicates *p*<0.05, **indicates *p*<0.01.

More evidence was obtained on bone histomorphometry to confirm the result. The H&E staining was performed on the sections of L1 vertebra. It was found that the average surface area of bone trabeculae dramatically decreased in WT.OVX mice compared with WT.Sham mice (Figure 2A, left, 2B). However, there is no significant difference in the ratio of surface area of bone trabeculae to that of L1 vertebrae of *APN* KO mice between sham and OVX operations (Figure 2A, right, 2B).

### Deficiency of APN Results in Increased *ALP* Expression and Activity

**Figure 3:**
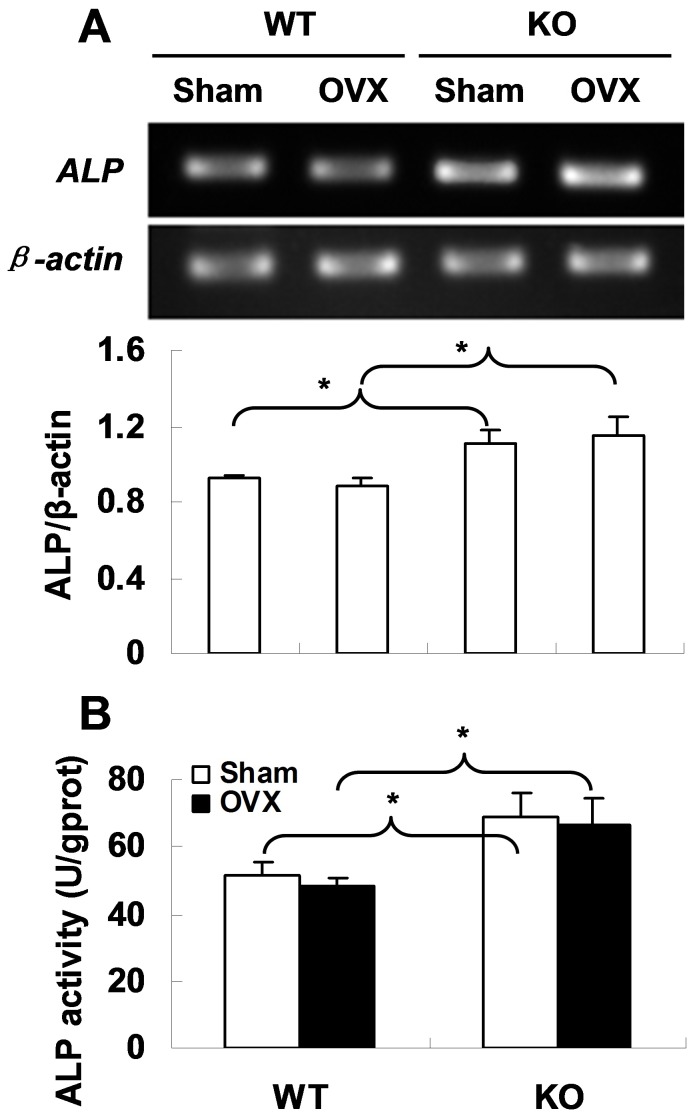
Increased *ALP* expression and activity in *APN* KO mice. (A) RT-PCR shows *ALP* expression in mouse vertebrae of each in four groups (top) and the band density was scanned and calculated (bottom). All reactions were repeated independently more than three times. (B) Increased ALP activity in vertebrae of *APN* KO mice. ALP activity was normalized to the amount of protein assayed. *indicates *p*<0.05.

To find out why APN deletion is associated with protection of bone mass, we evaluated the osteoblast activity in mouse vertebrae by testing *ALP* gene expression and protein activity. The RT-PCR showed a higher *ALP* mRNA level in *APN* KO mice as compared to WT mice (Figure 3A). Consistently, ALP activity was found significantly increased in KO mice than that in WT mice (*p*<0.05, Figure 3B). These findings indicate that APN deficiency could benefit osteogenesis in mice. Interestingly, we found that OVX surgery had no effect on ALP expression and activity in both WT and *APN* KO mice (Figure 3A, 3B), suggesting that OVX-induced bone loss could be caused by other factors rather than impaired osteogenesis.

### Deficiency of APN Results in Increased Osteoclastogenesis

**Figure 4:**
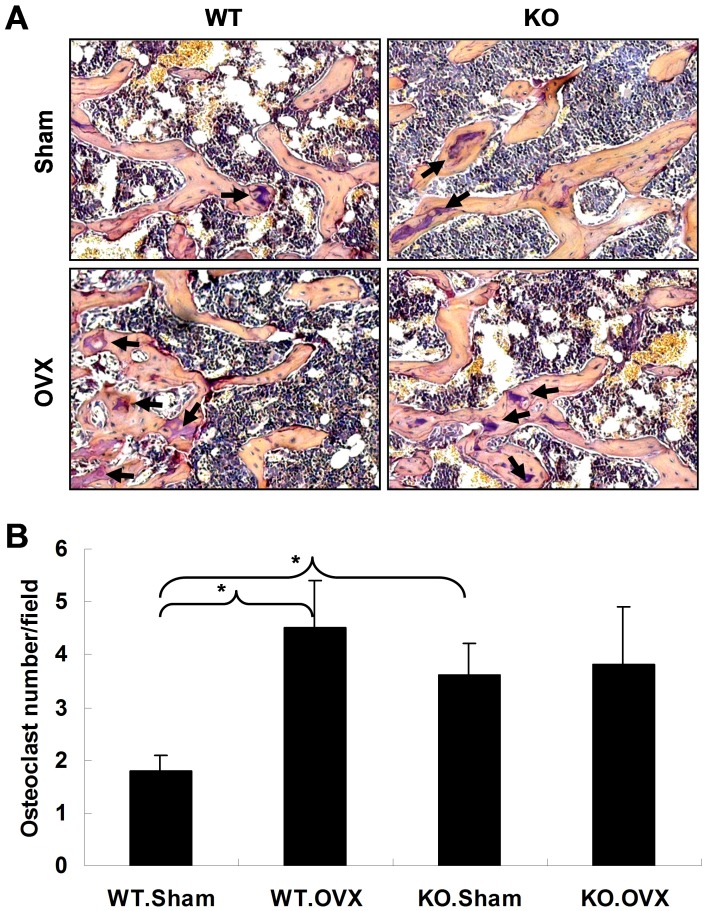
OVX surgery induces osteoclast formation. (A) Femora sections were stained with tartrate resistant acid phosphatase (TRAP) for osteoclasts (arrows). Original magnification 200×. (B) Average number of osteoclasts on each section are shown. The results are expressed as mean ± SD (n = 3). *indicates *p*<0.05.

Osteoclasts in femora were visualized by tartrate-resistant acid phosphatase (TRAP) staining. As shown in figure 4A, 4B, OVX induced a significant increase in osteoclast number in the femora of WT mice when compared to that in the Sham mice. But there was no significant difference in osteoclast number between Sham and OVX mice deficient for APN. Furthermore, the number of osteoclast cells in KO mice with either sham or OVX was augmented in comparison to that of WT sham. These data suggest that APN deficiency may increase bone loss by increasing osteoclast cell number, and APN may be involved in mediating OVX-induced bone loss.

### Deficiency of APN Enhances MSC Osteoblastic Differentiation and Consequent Extracellular Matrix Calcification

**Figure 5:**
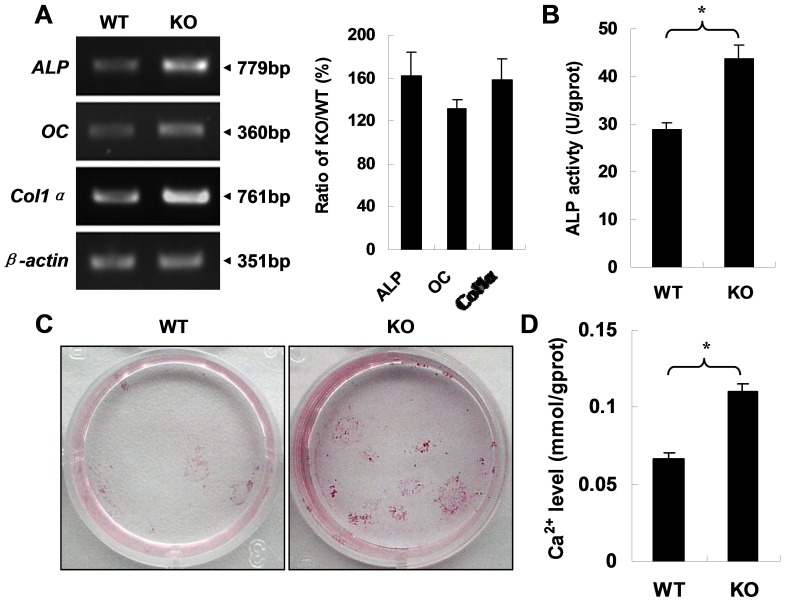
Increased osteogenic differentiation and extracellular matrix calcification in MSCs from *APN* KO mice. (A) RT-PCR shows that mRNA expression of the indicated genes (left). The respective ratio of band density (KO/WT, %) normalized with *β-actin* represents relative mRNA levels (right). A representative result of three independent experiments is shown. (B) Increased ALP activity in MSC lysate from *APN* KO mice. (C) Alizarin red S staining shows enhanced calcification ability of MSCs from *APN* KO mice (n = 3 for each). (D) Ca^2+^ content in cell lysate from *APN* KO mice is higher than that from WT mice (n = 3 for each). **indicates *p*<0.01.

To confirm the effect of adponectin on osteogenic differentiation and mineralization, mouse bone marrow derived mesenchymal stem cells (MSCs) were isolated from *APN* KO mice and their WT littermates since it is well documented that MSCs can be directed towards the osteogenic lineage in vitro when they are treated with dexamethasone, β-glycerophosphate, and ascorbic acid [18]–[22]. MSCs were cultured for 8 days and induced toward osteoblastic differentiation for 3 days. Total RNA was extracted from the cultures. The mRNA expression of three differentiation-related genes as osteoblastic markers (*ALP*, *osteocalcin* and *Col1α*) was detected using RT-PCR, and ALP activity was evaluated. The results showed that mRNA expression (Figure 5A) and ALP activity (Figure 5B) were upregulated in the cell lysate from *APN* KO mice. Consistent with these data, extracellular matrix calcification was enhanced in MSCs from *APN* KO mice (Figure 5C, 5D). All these observations suggest that deletion of APN promotes MSC osteogenic differentiation and consequent extracellular matrix calcification.

### APN Deficiency is Associated with Elevated MSC Proliferation and Expression of *Runx2* and *Osterix*

**Figure 6:**
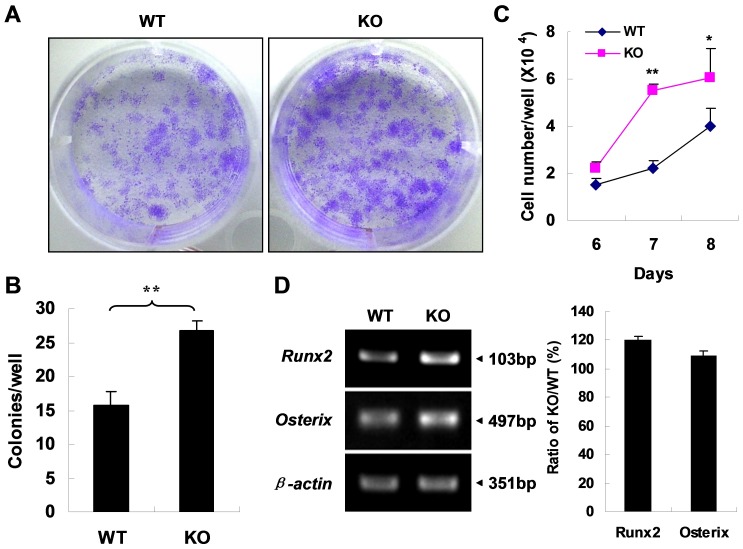
APN deficiency enhances MSC proliferation and osteoblastic differentiation. (A) Crystal violet staining shows an increased number of CFU-F in MSCs from *APN* KO mice. (B) The graphs indicate the number of positive colonies/well by crystal violet staining. (C) MSC proliferation assay by cytometry shows increased proliferation of *APN* KO MSCs. *indicates *p*<0.05, **indicates *p*<0.01. (D) RT-PCR shows that up-regulation of two transcriptional factors *Runx2* and *Osterix* in MSCs from *APN* KO mice. A representative result of three independent experiments is shown.

To further explore the mechanisms by which deficiency of APN promotes bone formation, the proliferation of MSCs from *APN* KO mice and WT littermates was evaluated. MSCs were isolated and cultured for 8 days followed by crystal violet staining (Figure 6A). The number of colonies was counted (Figure 6B). The data showed that MSCs deficient for APN showed higher proliferation than that of WT cells. To further confirm this result, the total number of MSCs at day 6–8 was counted by cytometry (Figure 6C). The result indicated that the number of *APN* KO MSCs increased faster than that of WT cells. These data suggest that ablation of self-produced adiponectin *in vitro* promotes proliferation of MSCs.

Moreover, total RNA was extracted from MSCs after 8 days of culture in ordinary medium. The RT-PCR results showed that two transcription factors, *Runx2* and *Osterix*, that are critical for the process of osteoblastic differentiation were up-regulated in MSCs from *APN* KO mice (Figure 6D). Thus, our results indicate that a possible mechanism for APN deficiency induced protection of osteoporosis could be through increased proliferation of MSCs and expression of *Runx2* and *Osterix*, thereby enhancing MSC osteoblastic differentiation and consequent extracellular matrix calcification.

## Discussion

Clinical studies that report significant negative correlations between circulating APN and bone mass [7], [8], [23]–[28], suggest that APN may exert negative effects on bone mass and that these effects may be mediated by menopausal status. In the present study, we investigated the role of APN in bone formation *in vivo* and *in vitro* using an *APN* KO mouse model. To mimic the internal environment of postmenopausal women, we performed OVX surgery on *APN* KO female mice. In agreement with the literature, we found that APN deficiency protects against OVX induced reduction of BMD and biomechanical strength properties at skeletal sites. In support of these findings, *in vitro* data showed that APN deficiency increases MSCs osteogenic proliferation, differentiation and consequent extracellular matrix calcification.

Much evidence shows that obesity is strongly correlated with increased bone mass [29]–[33] and that reductions in body weight are related to bone loss [34]–[36]. Therefore, it would be possible that APN, an adipocyte-derived hormone, negatively associated with obesity, could have negative effects on bone. However, in the current study, we did not find significant differences in body weight between the *APN* KO and WT mice before or after OVX surgery, suggesting that *APN* deletion protects against bone loss via other pathways that have nothing to do with body weight. Lots of studies show that body weight of OVX mice increases several weeks after surgery as compared to their sham control [37], [38]. In agreement with these reports, our results show that body weight of OVX mice augments in both KO and WT mice.

Previous studies have shown that the development of alkaline phosphatase-positive cells is inhibited in preosteoblast cultures by APN [5]. In agreement with this notion, Luo XH et al. found that APN inhibited ALP activity, osteocalcin secretion, Runx2 protein expression, and the formation of mineralized nodules [39]. The present study clarifies a direct effect of APN on osteoblast cells. Specifically, we have demonstrated the effect of APN on MSC proliferation, differentiation and mineralization. The bone-forming activity of osteoblasts is important in determining bone mass [40], [41]. Our study reveals that the mRNA expression and protein activity of ALP from lumbar and femora are significantly increased in *APN* KO mice over those in WT mice. These findings are further confirmed by the effect of APN on the osteoblastogenesis of MSCs through an autocrine pathway, supported by a significant increase in proliferation, differentiation and mineralization activity of MSCs of *APN* KO mice as compared with that of WT mice. These results are similar to those reported in the literatures [5], [39]. Many osteoblast differentiation transcription factors have been identified through human genetic studies as well as through mouse genetics and molecular studies [42]. Among those, Runx2 and Osterix are required for bone formation [43]. Runx2, a member of the Runt domain family of transcription factors, has been shown to be the earliest and most powerful molecular determinant of osteoblast differentiation. Osterix is a Runx2-dependent osteoblast-specific transcription factor that is specifically expressed in osteoblasts of all skeletal elements [43]. Inactivation of Osterix in mice results in perinatal lethality owing to a complete absence of bone formation [43]. In the current study, the elevation of *Osterix* and *Runx2* expression could explain, at least in part, the mechanism of APN deficiency effects on bone formation.

Maintenance of bone mass and integrity requires a tight balance between resorption by osteoclasts and formation by osteoblasts. Our results indicate that there is a much higher ALP activity, as well as a larger amount of osteoclasts in KO mice, compared with WT mice. This might explain why there is no significant difference in BMD between KO and WT mice without surgery. When performed surgery, osteoclasts in femora increased remarkably in WT.OVX mice, compared with that of WT.Sham mice. However, no increase has been found in KO mice when performed OVX surgery. The increased osteoclasts in WT.OVX mice may lead to enhanced bone resorption which results in significant loss of bone mass. While in *APN* KO mice, the increased osteogenesis might compensate the enhanced bone resorption induced by OVX.

Collectively, the data from our study indicate that APN deficiency can protect against OVX induced osteoporosis in mice. In this study, we have shown that APN deficiency has positive effect on both bone formation and resorption, reflecting a complex role of adiponectin in regulating bone metabolism. This could be the reason, at least in part, why no obvious bone phenotype was observed in several adiponectin knock-out mouse lines and adiponectin overexpressing transgenic mouse lines [5], [12]. In fact,the role of APN on BMD regulation remains controversial in the previous studies including clinical association, animal models, and *in vitro* studies [3]–[12]. Although we didn’t evaluate the MSC osteoclastic differentiation and proliferation in the absence of APN *in vitro*, we found that APN deficiency may increase bone loss through promoting osteoclastogenesis evidenced by increased osteoclast cell number *in vivo*. This finding is similar to previous reports [5], [44]–[46]. Since we showed that APN deficiency inhibits OVX-induced bone loss and promotes osteoclastogenesis, it is very possible that increased osteoblastic activity exerts an important role in the process of bone remodeling. Therefore, we focused our research on bone formation and found APN deficiency increases osteoblastic activity by enhancing MSC osteoblastic differentiation and consequent extracellular matrix calcification.

We have shown that APN deficiency exerts positive effect on both osteoblast and osteoclast activity in the process of OVX-induced osteoporosis in mice. It seems that APN plays a role in regulating bone metabolism through more complicated mechanism. Actually, more and more evidences suggest that the molecular adipokine-bone interactions should be considered in the context of different pathology status or diseases such as obesity and metabolic syndromes. Besides adiponectin, various biologically active molecules, such as estrogen, resistin, leptin, insulin, preptin and some proinflammatory cytokines, not only affect energy homeostasis, but are also involved in bone metabolism and, thus, contribute to the complex cross-talk between fat mass and bone [44]. This would be helpful for better understanding the contradictory effect of adiponectin on bone metabolism reported by different studies. In this study, we found that adiponectin deficiency is of advantage to regulating the balance between bone formation and bone resorption after OVX surgery and protects against OVX-induced osteoporosis in mice. This suggests that targeting APN could be therapeutically beneficial for patients with reduced bone mass, especially for postmenopausal women’s osteoporosis. Although APN shows a complex biology, reduced APN is also associated to greater cardiometabolic risk.
